# Supplementary material for: Glucocorticoid receptor signalling activates YAP in breast cancer
Source: Nat Commun. 2017 Jan 19;8:14073. doi: 10.1038/ncomms14073 (PMC5253666; doi:10.1038/ncomms14073)
Supplement: Supplementary Information — Supplementary Figures and Supplementary Tables [file ncomms14073-s1.pdf]

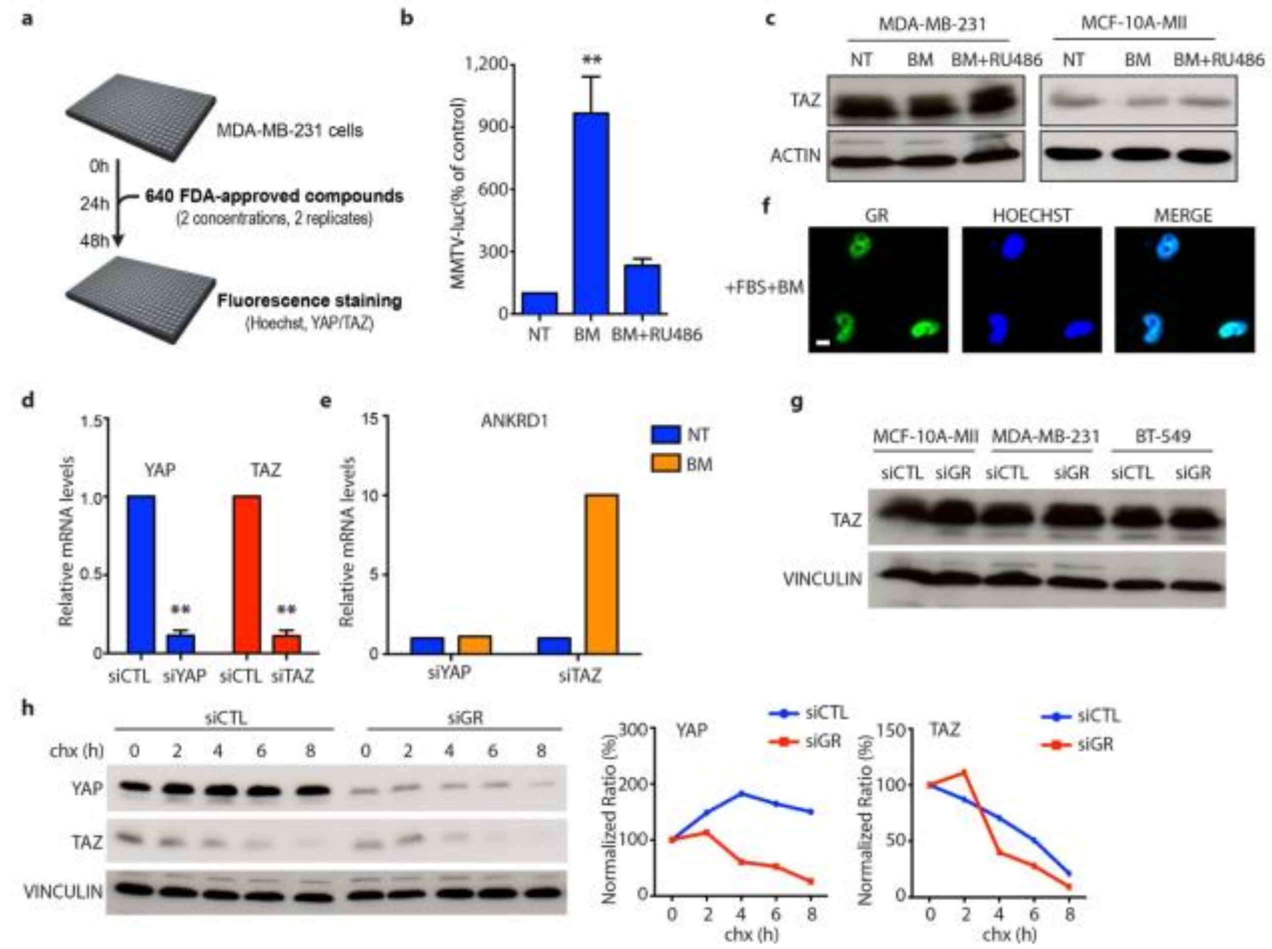

Supplementary Figure 1. **(a)** Schematic representation of the high-content screening. MDA-MB-231 cells were seeded in 384-well plates and 24h later the FDA-approved compounds were added to cells at 1 and 10 µM. 24h after treatment, cells were fixed and processed for immunofluorescence for YAP and stained with Hoechst. Automated image acquisition and analysis was then performed to analyse the fluorescence intensity. The screening was performed in duplicate; ca. 4,500 cells were analysed per experimental condition and replicate. **(b)** Luciferase reporter assay (MMTV-luc). MDA-MB-231 cells were treated with Betamethasone (BM) 1µM alone or in combination with RU486 1µM for 24h. Data are normalized to NT. Error bars represent mean ± s.d., from *n*=3 biological replicates. **(c)** MDA-MB-231 and MII cells were treated with Betamethasone (BM) 1µM alone or in combination with RU486 1µM for 24h. Representative blots are shown. n=3. **(d)** qRT-PCR analysis of MDA-MB-231 cells relative to Figure 1D. Error bars represent mean ± s.d., from *n*=3 biological replicates. **(e)** qRT-PCR analysis of MDA-MB-231 cells transfected with indicated siRNA for 48h and treated with 1µM Betamethasone (BM) for 24h. CTL siRNA is control siRNA. **(f)** Representative images of immunofluorescence in MDA-MB-231. Cells were grown in 10%FBS and treated with 1µM Betamethasone (BM) for 24h. Scale bars, 15 µm. **(g)** MII, BT-549 and MDA-MB-231 cells were transfected with control (siCTL) or Glucocorticoid Receptor (siGR) siRNA for 48h. Representative blots are shown. **(h)** MDA-MB-231 cells were transfected with control (siCTL) or Glucocorticoid Receptor (siGR) siRNA for 48h and treated with cycloheximide 50µM for indicated hours. Representative blots (left) and densitometry analysis (right) are shown. $*P < 0.05$, $**P < 0.01$; Two-tailed Student's *t* -test is used throughout.

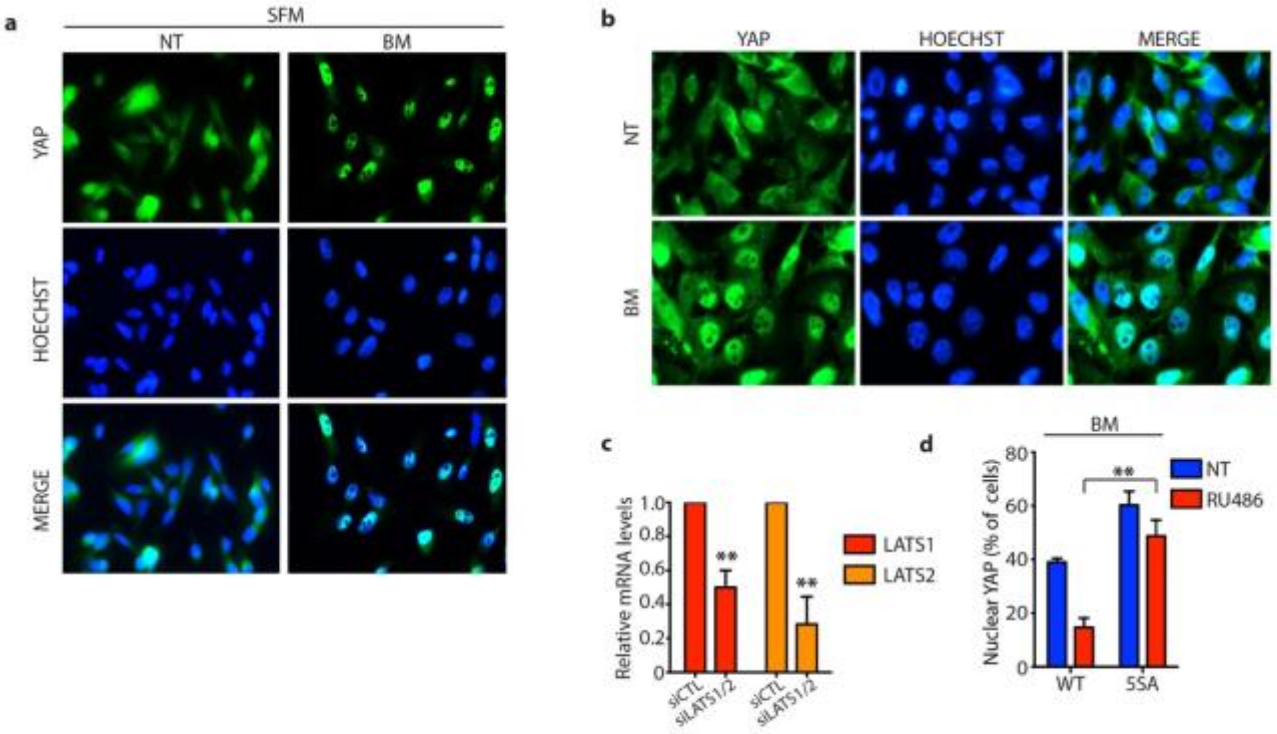

Supplementary Figure 2. **(a)** Immunofluorescence images shown in Figure 3C, here presented with their nuclear staining (Hoechst). **(b)** Immunofluorescence images shown in Figure 3E, here presented with their nuclear staining (Hoechst). **(c)** qRT-PCR analysis in MDA-MB-231 transfected with indicated siRNAs. Error bars represent mean ± s.d., from n=3 biological replicates. **(d)** Quantification of cells with nuclear YAP by immunofluorescence. MDA-MB-231 transiently overexpressing YAP-WT or YAP-5SA were treated with RU486 1μM for 24h in serum-free medium containing BM 1μM. Error bars represent mean ± s.d., from *n*=3 biological replicates. *$P < 0.05$, **$P < 0.01$; Two-tailed Student's *t* -test is used throughout.

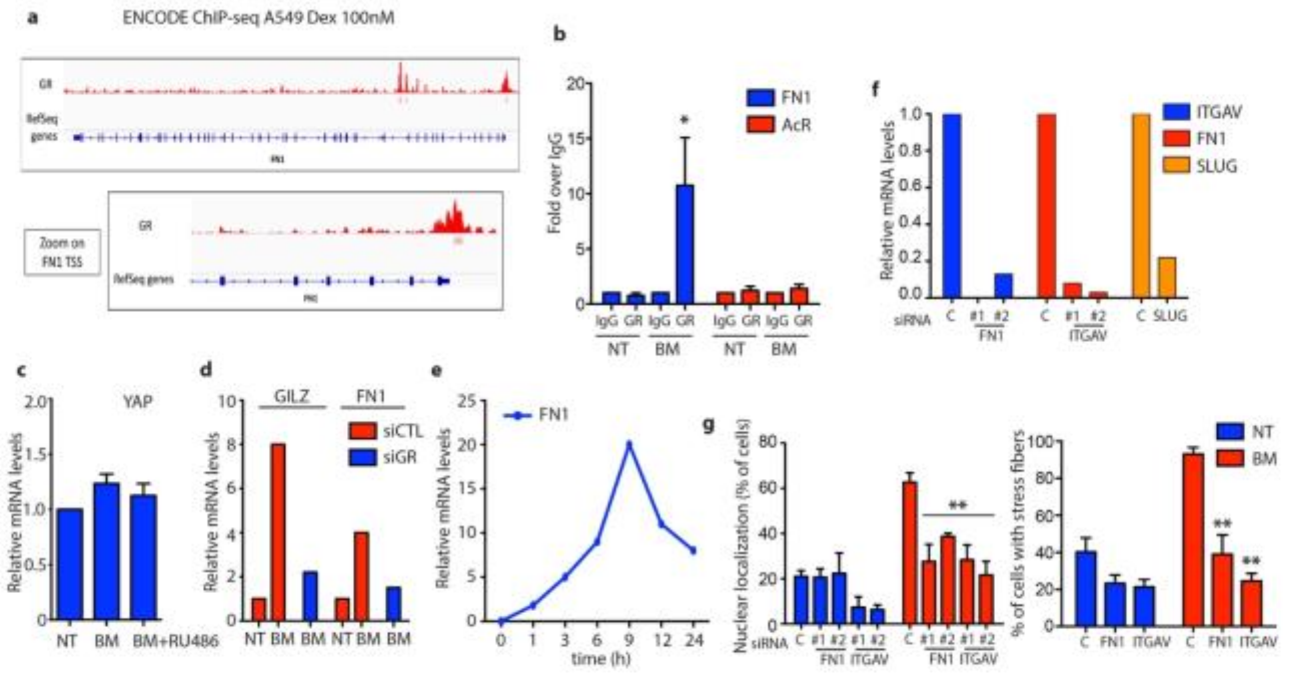

Supplementary Figure 3. (a) ChIP-Seq data for GR in A549 cell line treated with 100nM Dexamethasone from ENCODE project. Signal represents normalized read density of GR; peaks are regions with significant binding**. (b)** Upon 6h of betamethasone 1μM treatment, MDA-MB-231 cells were subjected to ChIP with either anti-GR antibody or normal Rabbit IgG. Binding of GR to the FN1 promoter was quantified by calculating the fold increase of normalized immunoprecipitated chromatin over the control IgG by qRT-PCR. The amplification of genomic region of the muscarinic acetylcholine receptor (AcR) promoter was used as control of GR specificity binding. Error bars represent mean ± s.d., from n=3 biological replicates. **(c)** qRT-PCR analysis of MDA-MB-231 treated with 1μM Betamethasone (BM) alone or in combination with RU486 1μM for 24h. **(d)** qRT-PCR analysis of MDA-MB-231 transfected with indicated siRNA for 48h and treated with 1μM Betamethasone (BM) alone or in combination with RU486 1μM for 24h. **(e)** qRT-PCR analysis of serum starved MDA-MB-231 treated with 1μM Betamethasone (BM) alone or in combination with RU486 1μM for the indicated times. **(f)** qRT-PCR analysis in MDA-MB-231 transfected with indicated siRNAs. Error bars represent mean ± s.d., from n=3 biological replicates. **(g)** Quantification of cells with nuclear YAP (left) or with stress fibers (right) by immunofluorescence. MDA-MB-231 cells were transfected with indicated siRNAs for 24h and treated with BM 1μM for additional 24h in serum-free medium. Error bars represent mean ± s.d., from n=3 biological replicates. $*P < 0.05$, $**P < 0.01$; Two-tailed Student's *t* -test is used throughout.

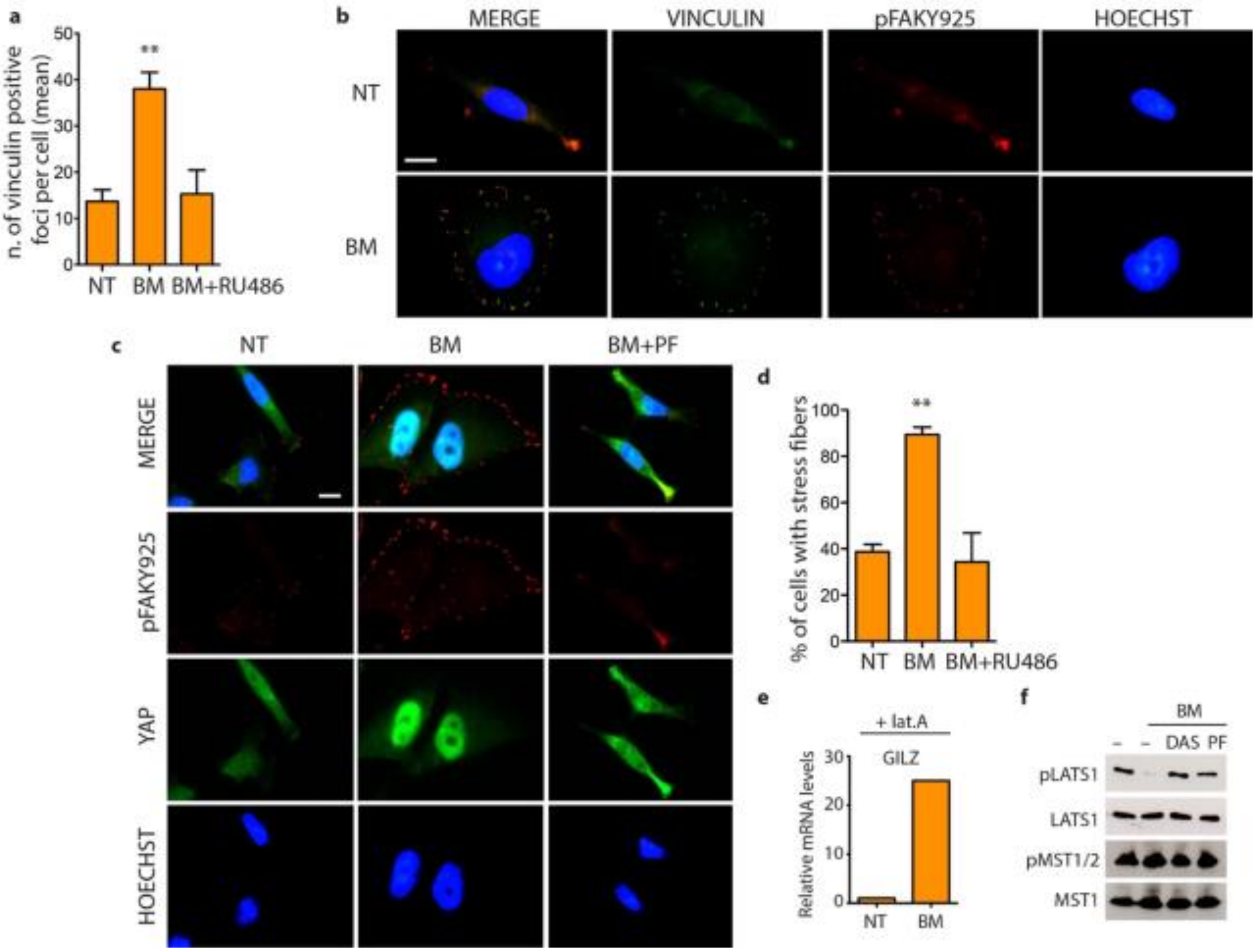

Supplementary Figure 4. **(a)** Quantification of vinculin positive foci by immunofluorescence in MDA-MB-231 cells treated as indicated for 24h in serum-free medium. Error bars represent mean ± s.d., from n=3 biological replicates. **(b)** Representative images of immunofluorescence in MDA-MB-231. Cells were grown in serum free medium and treated with Betamethasone (BM) 1 μM for 6h. Scale bars, 15 μm. **(c)** Representative images of immunofluorescence in MDA-MB-231 relative to Figure 5C. **(d)** Quantification of cells with stress fibers by immunofluorescence. MDA-MB-231 cells were treated as indicated for 24h in serum-free medium. Error bars represent mean ± s.d., from n=3 biological replicates. **(e)** qRT-PCR analysis of MDA-MB-231 cells grown in serum-free medium containing latrunculin A. 0.5 μM (lat.A) and treated with Betamethasone (BM) 1 μM for 24h. Error bars represent mean ± s.d., from n=3 biological replicates. **(f)** MDA-MB-231 cells were grown in serum-free medium in presence of Betamethasone (BM) 1 μM alone or in combination with Dasatinib (DAS) 0.1 μM or PF-573228 (PF) 5μM for 24h. Representative blots are shown. *$P < 0.05$, **$P < 0.01$; Two-tailed Student's t-test is used throughout.

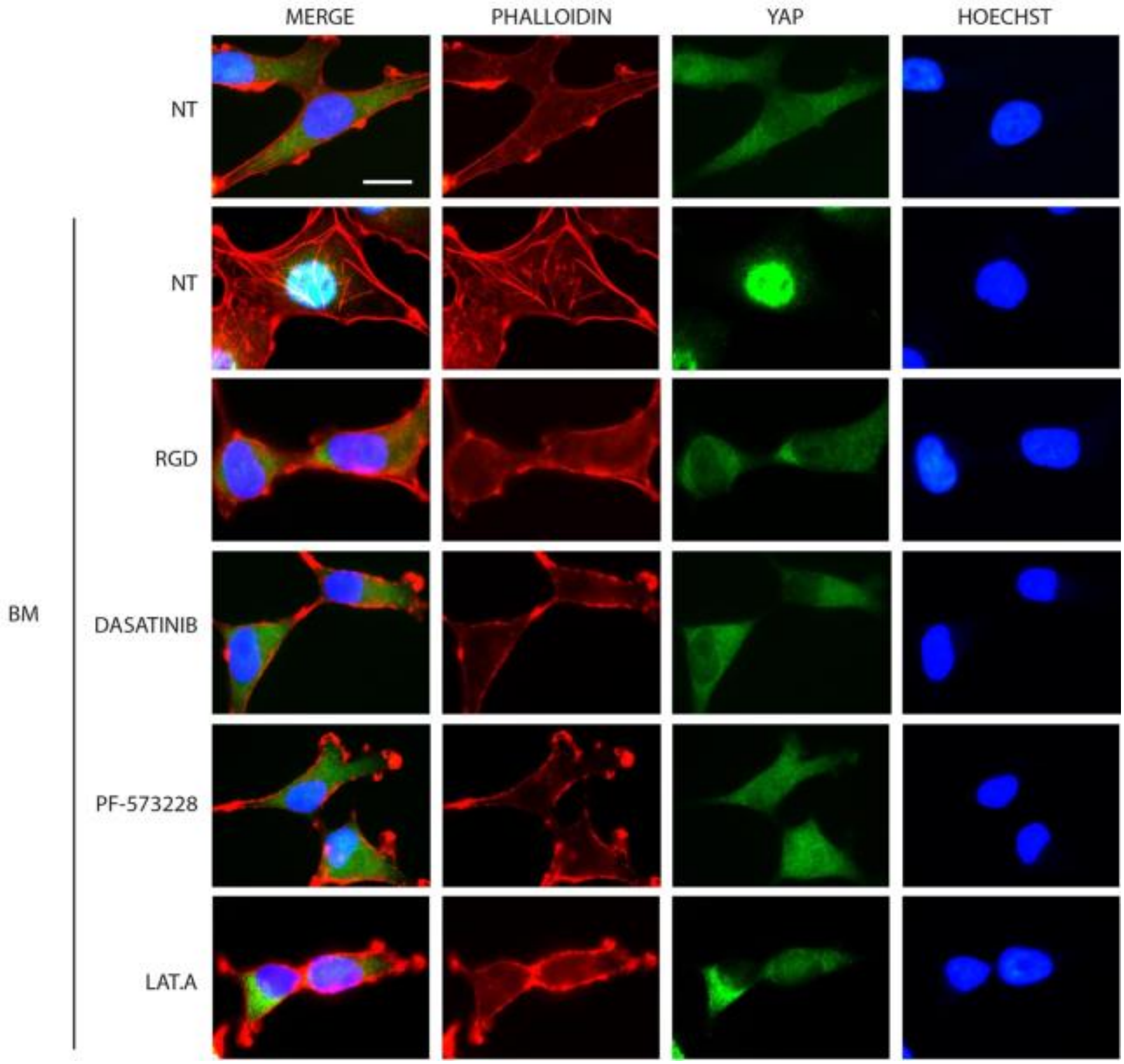

Supplementary Figure 5. Representative images of immunofluorescence in MDA-MB-231. Cells were grown in serum free medium containing Betamethasone (BM) 1µM and treated as indicated for 6h. LAT. is latrunculin A. 0.05 µM. Scale bars, 15 µm.

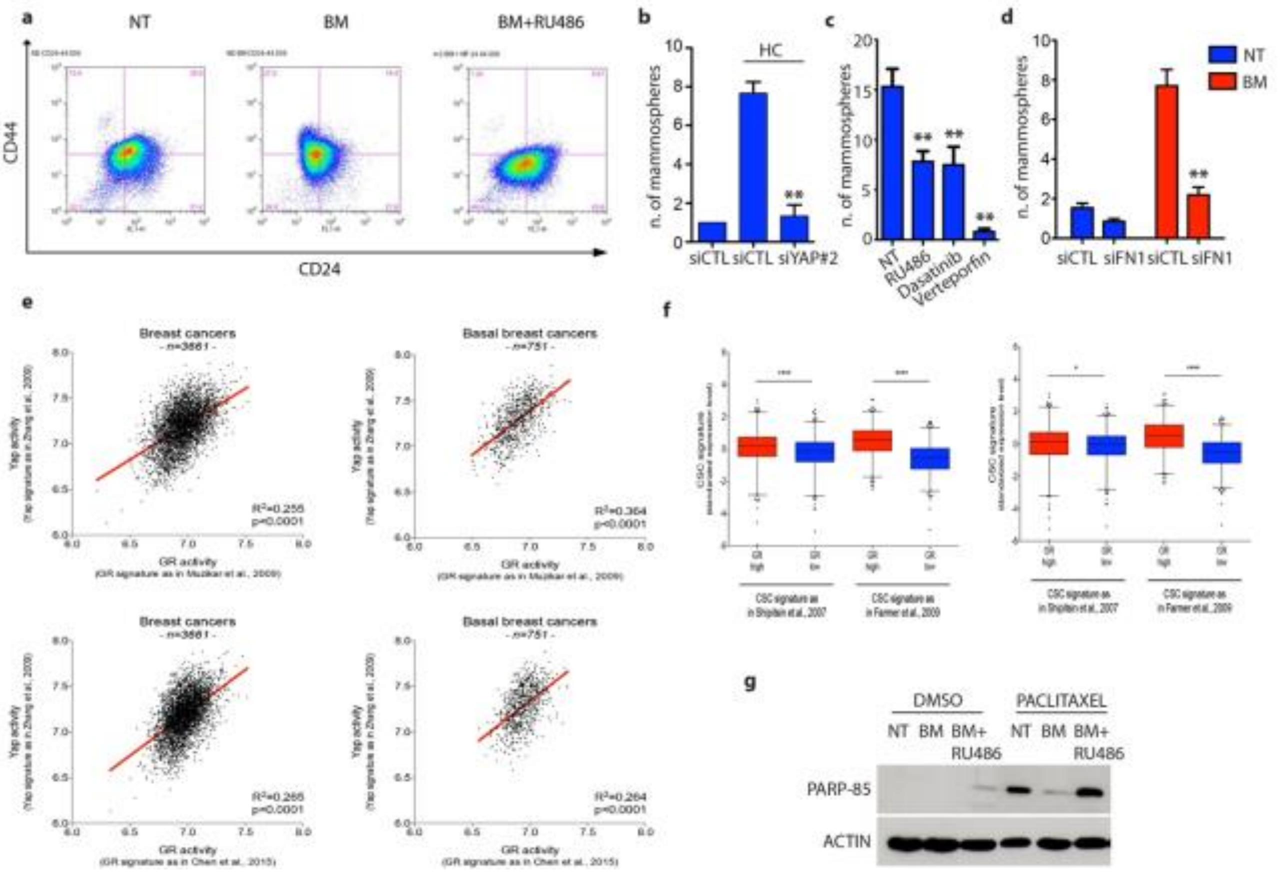

Supplementary Figure 6. **(a)** CD44/24 FACS analyses of MII cells treated as indicated for 5 days. Percentage of CD44/CD24 cell populations is indicated. **(b)** Number of secondary mammospheres from MDA-MB-231 cells as in Figure 6b. Error bars represent mean ± s.d., from *n*=3 biological replicates. **(c)** Number of secondary mammospheres from MDA-MB-231 cells as in Figure 6h. Error bars represent mean ± s.d., from *n*=3 biological replicates. **(d)** Number of secondary mammospheres from MDA-MB-231 cells as in Figure 6b. Error bars represent mean ± s.d., from *n*=3 biological replicates. **(e)** Scatter plot (black dots) and linear regression (red line) of standardized expression values indicate a positive correlation between gene signatures of GR activity (upper panels, [42]; lower panels, [26]) and a gene signature denoting YAP activity [75], in a metadataset of n = 3,661 primary human breast cancers and in the n = 751 PAM50 basal samples (see Materials and Methods). **(f)** Primary human breast cancers (n=3,661) of a metadataset were stratified according to high or low GR activity and then the levels of the breast stem cell signature scores [52,53] were determined in the two groups. GR activity scores were obtained summarizing the standardized expression levels of signature genes into a combined score with zero mean [72]. Stem cell signature levels have been calculated as the standardized average expression of all signature genes in high and low sample subgroups (see Materials and Methods). Stem cell signature level is significantly higher in tumours with high levels of GR activity, as visualized by the box plot (*P < 0.05, ****P < 0.0001, two-tailed Student's t-test). **(g)** MDA-MB-231 cells were treated with vehicle (NT) or Betamethasone 1 μM (BM) alone or in combination with RU486 1μM, and DMSO or Paclitaxel 0,1 μM for 48h. Representative blots are shown. **P* < 0.05, ***P* < 0.01; Two-tailed Student's *t* -test is used throughout.

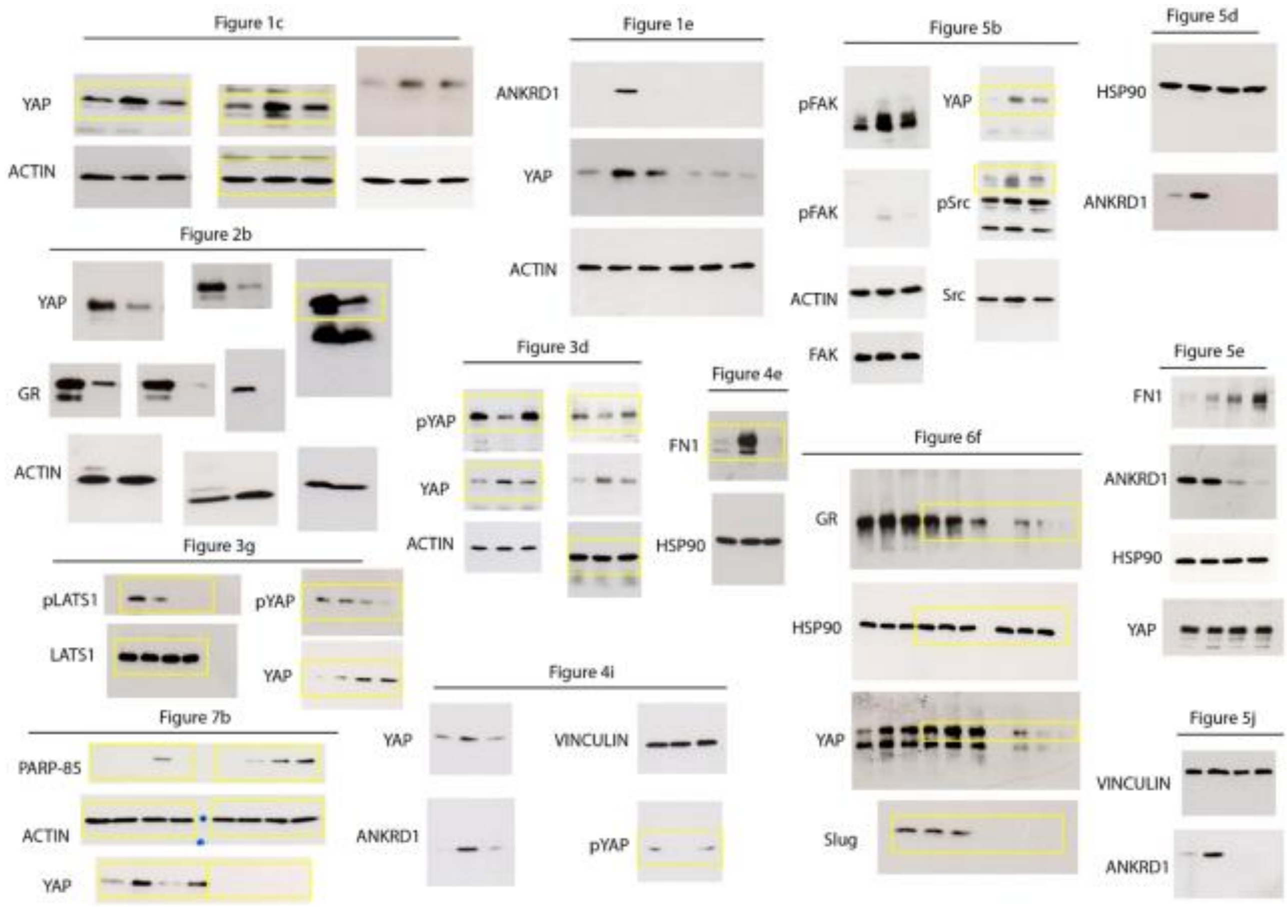

Supplementary Figure 7. Uncropped blots of main figures.

Muzikar et al., 2009				*Chen et al., 2015*		
CDKN1C	SH3TC1	SDK2	AKAP13	ABLIM3	LHFPL2	TLR2
DNAJC15	MT2A	SRGN	NR1D2	ABTB2	LHX6	TMEM164
TFCP2L1	SCNN1G	KIAA1462	EMP2	ACSL1	LIFR	TNFAIP3
FKBP5	SPIDR	CTGF	SIX3	ADAMTS14	LINC00341	TNFAIP8L3
FKBP5	PDK4	HIPK2	TNFAIP3	ADAMTS9	LINC01085	TNS2
FKBP5	MT1H	MCL1	MAOA	ADRA1B	LRRC8A	TOP1
RRAD	FOXO3	ZBTB20	ALOX5AP	AFAP1L1	MAOA	TOP1P1
FGD4	ZBTB20	ZBTB20	FLVCR2	AGFG2	MIR503HG	TPRA1
RRAD	ABHD2	PER2	KLHL29	AKAP13	MT1E	TRNP1
TSC22D3	SCNN1A	STARD13	KIAA0232	ALOX15B	MT1X	TSC22D3
CDKN1C	SERPINE1	PDK4	AKAP13	ALPP	MT2A	TSC22D4
CIDEC	AKAP13	NA	CALD1	AMMECR1	NAGS	TSPYL2
FGD4	SOCS1	ENTPD2	ZC3H12A	ANGPTL4	NEDD4	TXNIP
PTGER4	ANPEP	IRAK3	FAM222B	ANKRD1	NEDD9	UBALD2
EDN3	CHST7	THBS1	OR7E14P	AQP3	NFIL3	VCL
CDKN1C	EPB41L4B	F2RL1	PHF20	ARHGEF26	NFKBIA	VSTM2L
FAM43A	NA	TRIM29	RASSF4	ARL4D	NREP	WDR60
UNC5B	NA	KLF9	NNMT	ARMC8	NT5DC3	XKRX
CDKN1C	SLC26A2	SLC19A2	ACACB	ARRDC2	NUDT16	ZBTB7B
FAM105A	CALD1	PACSIN2	ZNF281	B3GNT5	OSBPL5	ZDHHC8P1
METTL7A	CALD1	LIFR	TMEM43	BAIAP2	OSGIN2	ZFHX3
ANGPTL4	BAIAP2	EMP2	LINC01137	BATF	PACSIN2	ZFP36
SYBU	MCL1	ARRB1	FBRSL1	BCL6	PDLIM1	ZFP36L2
PRR15L	CEBPD	KIAA0232	NOL3	BIN1	PGF	ZNF114
RGS2	GADD45A	LRRC8A	COBLL1	BIRC3	PIM3	ZNF57
CDKN1C	FAM105A	LIFR	TBX3	C6orf132	PKP2	ZNF608
TFCP2L1	ERRFI1	KLF6	DDIT4	CALCR	PLEKHA7	
CORO2A	GPR115	HPCAL1	AKAP12	CAMK2N1	PLIN2	
ANGPTL4	EMP1	PYGB	KCNG1	CDK5R1	PLXNA2	
RASSF4	ETNK2	THBS1	EMP2	CDKN1A	PNMT	
GMPR	EPB41L4A	NA	LIFR	CDKN1C	PPAP2B	
ST3GAL1	ARRB1	SLC22A5	GPR115	CEBPB	PRKX	
GRAMD4	DUSP1	KLF6		CEBPD	PTPN1	
ACSL1	NA	AHNAK		CNKSR3	PTPN13	
ZFP36	EMP1	ITGB4		CPD	PXYLP1	
CDH16	ARRB1	JPH2		CSF3	RAB20	
RGCC	NA	EPB41L4A		CTDSPL	RAB3IL1	
MT1X	ARRB1	KIAA1462		EDN1	RASSF4	
AKAP13	TIPARP	AQP3		EDN2	RCOR3	
SDPR	NEXN	SLC45A4		ELL2	RERE	
ACSL1	EPB41L4A	KLF4		EPB41L4B	RGCC	
PER1	NEURL1B	SHROOM3		EPSTI1	RGS19	
DUSP1	EPB41L4B	TMEM43		ERRFI1	RGS2	
TSC22D3	ITGB4	TANC2		ESYT2	RHOB	
FBXL16	PRKCD	ST3GAL1		EVA1C	RHOU	
SDPR	BEST2	PER1		FAM46B	RIPK2	
IGFBP1	RBM20	ITGB4		FGD4	RPRD1B	
PPARGC1B	B3GNT5	NA		FKBP5	S1PR1	
MT1X	SLC45A4	PIM3		FLVCR2	SAA1	
AKAP13	MAOA	SLC26A2		FOXO1	SCNN1A	
CDC42EP3	ARRB1	CPEB4		FOXO3	SDPR	
STOM	SERPINE1	RHOU		FPR1	SEC14L1	
PLEKHA7	CORO2A	S100P		FSTL3	SEC14L2	
CDC42EP3	BAIAP2	RASSF9		GADD45B	SERPINE1	
THBD	IL6R	NFKBIA		GATSL3	SGK1	
ARRB1	ANKRD44	SPX		GCNT1	SGPP2	
REEP1	EMP1	PHF20		GGT5	SH3RF3	
THBD	HIPK2	TMEM43		GJB3	SH3TC1	
CDC42EP3	NA	C1orf116		GLIS3	SHROOM2	
RAB11FIP1	RHOBTB2	RHOBTB2		GLUL	SIX2	
ABHD2	CDC42EP3	HIPK2		GPR153	SLC17A5	
ABHD2	MOB3B	TENC1		GRAMD3	SLC19A2	
IL6R	TNS4	RHOB		HELZ2	SLC20A1	
STOM	PLEKHA2	PPL		HS6ST1	SLC26A2	
FLVCR2	PACSIN2	IFNGR1		IGFBP7-AS1	SLC29A3	
PKP2	PER1	MOB3B		IL1R1	SLC38A2	
FOXO3	NAV2	ABHD2		IP6K2	SLC46A3	
NEXN	CITED2	BIRC3		IRS2	SLC7A2	
FOXO3	RAB11FIP1	PPARGC1B		IRX3	SMIM3	
KCNB1	TBC1D8	NOL3		ITGA10	SNAI2	
BIRC3	PRRG4	HSD11B2		ITGA5	SNORD49A	
HIPK2	RHOB	TACC1		ITPKA	SORBS2	
THBD	IFNGR1	APOL2		ITPKC	SP110	
KIAA1462	FOS	NEDD4		JADE1	SP6	
SLC4A11	NDRG1	TMEM164		JADE2	SPRY4	
NA	CITED2	NA		JPH2	SRGN	
AKAP13	SEC14L2	IRS2		KDR	ST3GAL1	
CALD1	ANKRD44	SLC4A11		KIAA1211L	ST3GAL6	
SNAI2	TBX3	RHOU		KIAA1551	ST5	
MT1F	CKB	CIART		KIF13B	SYNE3	
LOC283278	KLF6	ARRDC1		KLF13	TBC1D12	
ALPP	NA	MAN1C1		KLF15	TBC1D8	
FGD4	TFCP2L1	KLF6		KLF4	TFCP2L1	
MT1HL1	SHROOM3	MCL1		KLF7	THBD	
ARRB1	MT1G	TMEM164		KLF9	THRA	
CEBPD	MT1F	IRS2		KLHL4	TIPARP	

Supplementary Table 1: genes of the GR activity signatures

GENE	siRNA NAME	siRNA SEQUENCE
Human YAP1	siYAP #1	CUGGUCAGAGAUACUUCUU
Human YAP1	siYAP #2	GACAUCUUCUGGUCAGAGA
Human LATS1	siLATS1	CACGGCAAGAUAGCAUGGA
Human LATS2	siLATS2	AAAGGCGUAUGGCGAGUAG
Human GR	siGR	CCGAGAUGUUAGCUGAAAU
Human Slug	siSLUG	UCCGAAUAUGCAUCUUCAGGGCGCCCA
Human FN1	siFN1 #1	GUGGUCCUGUCGAAGUAUU
Human FN1	siFN1 #2	ACAAUGGAGUGAACUACAATT
Human ITGAV	siITGAV #1	UGAACUGCACUUCAGAUAUUU
Human ITGAV	siITGAV #2	CCAUGUAGAUCACAAGAUA

Supplementary Table 2: siRNAs sequences.

GENE	PRIMER NAME	PRIMER SEQUENCE
H3	H3 F	GTGAAGAAACCTCATCGTTACAGGCCTGGT
	H3 R	CTGCAAAGCACCAATAGCTGCACTCTGGAA
CTGF	CTGF F	AGGAGTGGGTGTGTGACGA
	CTGF R	CCAGGCAGTTGGCTCTAATC
ANKRD1	ANKRD1 F	CACTTCTAGCCCACCCTGTGA
	ANKRD1 R	CCACAGGTTCCGTAATGATTT
GR	GR F	TACCCTGCATGTACGACCAA
	GR R	TCCTTCCCTCTTGACAATGG
CYR61	CYR61 F	AGCCTCGCATCCTATACAACC
	CYR61 R	TTCTTTCACAA GGCGGCACTC
GILZ1	GILZ1 F	TCTGCTTGGAGGGGATGTGG
	GILZ1 R	ACTTGTGGGGATTCGGGAGC
LATS1	LATS1 F	CTCTGCACTGGCTTCAGATG
	LATS1 R	TCCGCTCTAATGGCTTCAGT
LATS2	LATS2 F	ACATTCACTGGTGGGGACTC
	LATS2 R	GTGGGAGTAGGTGCCAAAAA
GAPDH mouse	GAPDH m F	ATCCTGCACCACCAACTGCT
	GAPDH m R	GGGCCATCCACAGTCTTCTG
CTGF mouse	CTGF m F	CTGCCTACCGACTGGAAGAC
	CTGF m R	CATTGGTAACTCGGGTGGAG

Supplementary Table 3: Primer sequences.

Term	PValue	Benjamini
hsa05200:Pathways in cancer	1,20E-04	0.017
hsa04920:Adipocytokine signaling pathway	3,30E-04	0.022
hsa05222:Small cell lung cancer	2,00E-03	0.089
hsa05215:Prostate cancer	3,10E-03	0.102
hsa04510:Focal adhesion	3,10E-03	0.084
hsa04910:Insulin signaling pathway	3,10E-03	0.071
hsa05220:Chronic myeloid leukemia	3,10E-03	0.061
hsa04520:Adherens junction	3,10E-03	0.064

Supplementary Table 4: Pathway enrichment analysis (KEGG) of the Dexamethasone-induced genes list (DAVID functional annotation).

ID	Gene Name
795442	baculoviral IAP repeat-containing 3
781132	catenin (cadherin-associated protein), beta 1, 88kDa
797879	collagen, type VI, alpha 3
816368	epidermal growth factor receptor
800291	fibronectin 1
778146	filamin B, beta (actin binding protein 278)
797639	integrin, alpha 10
799503	integrin, alpha 5 (fibronectin receptor, alpha polypeptide)
777632	integrin, beta 4
825374	kinase insert domain receptor (a type III receptor tyrosine kinase)
809283	phosphoinositide-3-kinase, regulatory subunit 3 (gamma)
821855	placental growth factor
784849	platelet-derived growth factor alpha polypeptide
772977	son of sevenless homolog 1 (Drosophila)
786559	son of sevenless homolog 2 (Drosophila)
781513	v-raf-1 murine leukemia viral oncogene homolog 1
788771	vasodilator-stimulated phosphoprotein
822191	vinculin

Supplementary Table 5: List of Dexamethasone-induced genes involved in focal adhesion.
